# The Investigation of Protein Profile and Meat Quality in Bovine *Longissimus thoracic* Frozen under Different Temperatures by Data-Independent Acquisition (DIA) Strategy

**DOI:** 10.3390/foods11121791

**Published:** 2022-06-17

**Authors:** Xia Li, Shuyi Qian, Feng Huang, Kaimin Li, Yu Song, Jiqian Liu, Yujie Guo, Chunhui Zhang, Christophe Blecker

**Affiliations:** 1 Key Laboratory of Agro-Products Quality and Safety Control in Storage and Transport Process, Ministry of Agriculture and Rural Affairs, Institute of Food Science and Technology, Chinese Academy of Agricultural Sciences, Beijing 100193, China; lixia05@caas.cn (X.L.); qianshuyi@caas.cn (S.Q.); fhuang226@163.com (F.H.); song_yuu@163.com (Y.S.); liujiqian@caas.cn (J.L.); guoyujie324@163.com (Y.G.); 2 Gembloux Agro-Bio Tech, Department of Food Science and Formulation, TERRA Research Centre, University of Liege, 5030 Gembloux, Belgium; christophe.blecker@uliege.be; 3 School of Biological Science and Medical Engineering, Beihang University, Beijing 100191, China; lkm13436478237@163.com

**Keywords:** protein profile, meat quality, freezing temperatures, data-independent acquisition (DIA)

## Abstract

The influence of freezing on the protein profile and quality traits in bovine *Longissimus thoracic* (LT) muscle was investigated by the data-independent acquisition (DIA) technique. Compared to fresh meat, a total of 262 proteins were identified as differential abundance proteins (DAPs) in four frozen groups (−12 °C, −18 °C, −38 °C, and −80 °C). According to the bioinformatics analysis, most of the DAPs in the significant Go terms and the KEGG pathway were structure proteins and enzymes. Proteome changes in the frozen bovine muscle at −12 °C and −18 °C were more significant than those at −38 °C and −80 °C. The result was consistent with the deterioration trend of the meat quality. The correlation analysis revealed that 17 proteins were correlated closely with the color, shear force, thawing loss, and cooking loss of the frozen meat, which could be used as putative biomarkers for frozen meat quality. MYO18A and ME3 are newly discovered proteins that are associated with frozen beef quality. In addition, CTTN and SERPINB6 were identified in frozen groups, which exhibited a significant inverse correlation with thawing loss (*p* < 0.01). These findings reveal the quality changes induced by freezing at the protein molecular level and provide new insights into the control of quality deterioration.

## 1. Introduction

As a protein source, beef is an essential part of the human diet, and the demand for it is increasing worldwide [1]. In China, beef consumption has increased by 55% over the last ten years, accounting for 11% of global consumption, making China the world’s fastest-growing beef importer [2,3]. However, due to its high protein content and nutritional value, beef is easily perishable and has a short shelf life. In this sense, freezing has been commonly adopted as the safest and most cost-effective method of meat preservation, used in both the meat industry and in household kitchens, and it also plays a vital role in both export–import and regional trade [4,5]. Furthermore, freezing effectively inhibits microbial, endogenous enzymes, oxygen, and heat-induced biochemical activity, making it ideal for extending meat shelf life [6,7]. Nevertheless, the quality deteriorates with the freezing, including discoloration, poor texture, a decrease in the water-holding capacity (WHC), etc., which has resulted in massive economic losses for the meat enterprise [8,9]. Therefore, an exhaustive understanding of the physical and chemical changes caused by freezing is critical for the meat industry.

Protein is the main component of meat. The changes in protein properties such as structure and functionality are often cited as the primary cause of frozen meat deterioration [10]. Proteomics techniques have been used over the last two decades to identify biomarkers and illuminate the molecular mechanism of the protein change associated with the development of meat quality [11], such as color [12], tenderness [13,14], and water-holding capacity in early postmortem and meat processing [15,16,17]. The iTRAQ method was used to analyze the changes in mud shrimp and razor clam muscle proteins during frozen storage [18,19]. Triosephosphate isomerase, troponin, and peroxiredoxin-6 were discovered as potential biomarkers for long-term frozen Hengshan goat meat using label-free technology based on high-throughput proteomics [20]. Two-dimensional gel electrophoresis (2-DE) was used to make a distinction between the fresh and frozen–thawed pork [21]. However, all these approaches are established on the well-known Date Dependent Acquisition (DDA) methodology, which has the major disadvantage of losing information on low abundance peptides for accurate quantification [22]. In contrast to the traditional methods, the emerging data-independent acquisition (DIA) strategy has provided a new dimensionality for unlabeled quantitative proteomics [16]. The DIA mode divides the full mass range into many successive windows and captures all precursors and fragments in each window while keeping the low abundant ions with great repeatability and quantitative accuracy [22]. DIA has been used successfully to study disease-related cell lines, tissue, plasma samples, and meat processing [16,22,23]. Temperature is the most fundamental prerequisite for freezing, and it significantly impacts the quality of frozen meat [7,8]. Unfortunately, there is no systematic investigation of proteome modifications and molecular mechanisms related to frozen beef’s physical and chemical properties from a temperature perspective using the DIA method.

Thus, DIA-coupled LC-MS/MS technology was used in this study to reveal the differential expressivity and bioinformatics changes of frozen-induced bovine muscle protein and to identify the potential biochemical markers related to frozen beef quality. These findings will help improve the quality of frozen beef by revealing more information about the internal mechanisms that cause the quality of bovine *Longissimus thoracic* (LT) to decrease at different freezing temperatures.

## 2. Materials and Methods

### 2.1. Sample Collection and Preparation

A total of nine bull (Simmental × Qinchuan, aged 48 months old, live weight 600 ± 25 kg) were collected from the slaughterhouse (Beijing Zhuochen Animal Husbandry Co., Ltd., Beijing, China). *Longissimus thoracic* (LT) muscle samples were removed from the left side of each cold carcass (2–4 °C) after 36 h postmortem and transported in the iceboxes within 4 h to the laboratory at the Institute of Food Science and Technology, Chinese Academy of Agricultural Sciences. After trimming the visible adipose and connective tissues, each LT muscle was cut into 15 identical parts. Each steak had a 5.0 cm thickness, a 3.0 × 3.0 cm cross-section, perpendicular to the fiber direction, and weighed 71.61 ± 2.25 g. Subsequently, every steak was wrapped in polyethylene film (O_2_ permeability of 23,000 ± 40% cm^3^/m^2^/24 h/atm, CO_2_ permeability of 102,000 ± 40% cm^3^/m^2^/24 h/atm, and a moisture permeability rate of 39 g ± 40%/m^2^/24 h/atm). The 15 steaks from each LT muscle were equally assigned to the fresh group (CON), and four freezing treatments (−12 °C ± 0.5 °C, −18 °C ± 0.5 °C, −38 °C ± 0.5 °C, and −80 °C ± 0.5 °C), and each group contained nine biological replicates. After the center temperature reached the desired level, meat samples were kept at that level for 48 h, and then thawed to a central temperature of 4 °C ± 0.5 °C and kept in balance for 24 h. Approximately 5 g of minced samples were immediately frozen in liquid nitrogen and stored at −80 °C for proteomics analysis. The others were performed for measurements of quality.

### 2.2. Meat Quality Determination

To assess meat quality, the color, shear force, thawing, and cooking loss were determined. The thawing loss was calculated by comparing the meat quality before and after thawing according to the method of [24]. The surface CIE (L*, a*, and b*) values were measured by a chroma colorimeter (CM-600D, Illuminant D65, 10⁰ viewing angle, 2.54 cm diameter aperture, Konica Minolta Sensing Americas Inc., Ramsey, NJ, USA). The lightness (L*), redness (a*), and yellowness (b*) were recorded in the manner recommended by [25]. Shear force values were determined using the C-LM3 digital meat tenderness meter (Tenovo International Co., Limited, Beijing, China) in accordance with [26]. In brief, each muscle sample was cooked inside the cooking bag in a water bath at 80 °C until the core temperature reached 72 °C, then cooled to room temperature. Before and after cooking, the difference in quality was used to calculate the cooking loss [26]. After that, six cores (1 × 1 × 2 cm) were removed from each treatment sample parallel to the muscle fiber direction. The cross-head speed of the knife blade was 5 mm/s. The output was listed in grams (g).

### 2.3. Protein Extraction and Digestion

**Figure 1:**
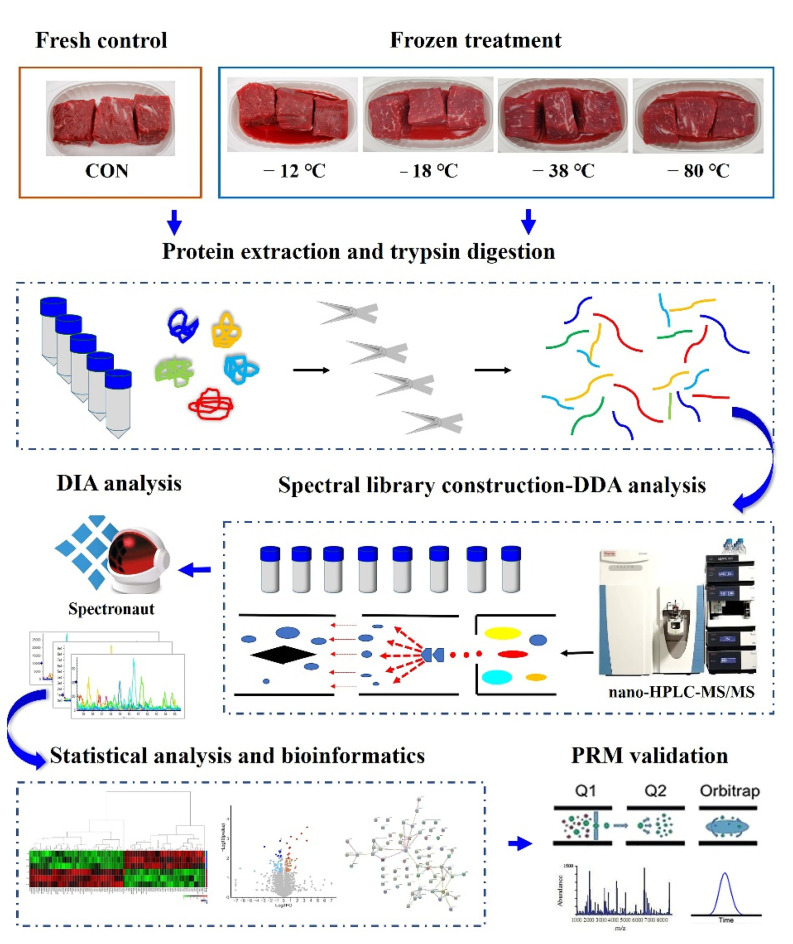
Experimental design and workflow for quantitative proteomic analysis of beef using DIA technology.

Figure 1 depicts the experimental design and workflow. DIA proteomics analyses were performed in the manner described by [16,23], with minor modifications. A sample pooling strategy was used for the proteomic study. Minced meat gathered from 3 individual LT muscles were combined as one replicate, with 3 replicates per treatment. Each pooled sample (1 g) was dissolved in the lysis buffer containing 1% SDS, 8.0 M urea, and 1 × protease inhibitor cocktail, vibrated and milled for 400 s, repeating this procedure three times, and then lysed for 30 min in an ice-cold bath. The supernatant was collected and stored at −80 °C after 15 min of centrifugation at 4 °C and 21,500× *g*. Protein concentration was determined via the BCA Protein Assay Kit (cat. no. P0010, Beyotime Institute of Biotechnology, Shanghai, China) following the manufacturer’s instructions.

Protein extracts (100 μg) were diluted to the final volume of 100 μL with 8 M urea. Then, the protein was reduced at 37 °C for 1 h using 2 μL 0.5 M Bond-Breaker TCEP Solution (cat. no. 646547-10* 1 ML, SIGMA, New York, NY, USA), and the cysteines were alkylated for 40 min at room temperature in the dark with 4 μL iodoacetamide (IAA, 1M). The proteins were then precipitated overnight at −20 °C using five portions of prechilled acetone. The solution was centrifuged at 12,000× *g* for 20 min at 4 °C, and the precipitates were washed with 1 mL 90% prechilled acetone before being redissolved in 100 μL of 100 mM TEAB solution (this step was repeated two times). Each sample was digested with trypsin (cat. no. V3155, Promega, Madison, WI, USA) in a 1:50 ratio and incubated overnight at 37 °C. The desalted peptide mixture was accurately measured using the PierceTM Quantitative Colorimetric Peptide Assay (cat. no. 23275, Thermo Fisher Scientific, Waltham, MA, USA) before being freeze-dried for further analysis.

### 2.4. Spectral Library Generation

#### 2.4.1. High-pH Reversed-Phase Separation

Tryptic-digested peptides were redissolved in 0.1% Trifluoroacetic acid (cat. no. 302031-100 ML, SIGMA) and then fractionated by high-pH separation using the Pierce High pH Reversed-Phase Peptide Fractionation Kit (cat. no. 84868, Thermo Fisher Scientific). A linear gradient of acetonitrile ranging from 5% to 50% was used to separate high pH solutions. Next, the eluted peptides were divided into 8 fractions and freeze-dried in a vacuum concentrator for the next step.

#### 2.4.2. Nano-HPLC-MS/MS Analysis for DDA and DIA Analysis

The analysis was performed using an online nanospray LC-MS/MS on an Orbitrap Fusion^TM^ Lumos^TM^ Tribrid^TM^ coupled with an EASY-nLC 1200 system (Thermo Fisher Scientific, MA, USA). For the DDA analysis, the peptide fractions were redissolved in mobile phase A (0.1% formic acid in water). First, 5 μL peptide solution was injected onto an analytical column (Thermo Fisher Scientific Acclaim PepMap C18, 75 μm × 25 cm) for separation at a flow rate of 250 nL/min at 40 °C. Then, the supernatant was eluted with a linear gradient from 4% to 95% mobile phase B (0.1% formic acid in acetonitrile) for 130 min. The MS parameters were set to operate one full scan from *m*/*z* 350 to 1500 with a resolution of 120,000, and the HCD-MS/MS scans were performed at a resolution of 30,000 with an isolation window of 4. The automatic gain control (AGC) value was set to 8 × 10^5^ for M and 5 × 10^4^ for MS/MS with a maximum injection time of 50 ms (MS) and 86 ms (MS/MS), respectively. The precursor ions were fragmented at collision energies of 25%, 30%, and 35%.

Concerning DIA analysis, 1 μL 10 × iRT peptides (Ki3002, Biognosys AG, Schlieren, Switzerland) were combined with redissolved solution (9 μL) and separated using the same nano-LC system and gradient as for DDA analysis. The mass spectrometer was set to DIA mode, which automatically alternated between MS and MS/MS modes. The full mass values ranged from 350 to 1200 m/z (AGC 1 × 10^6^, 100 ms maximum injection time), with a 120,000 resolution. In addition, HCD-MS/MS was programmed with a resolution of 30,000, an AGC target of 1 × 10^5^, and a collision energy of 33.

#### 2.4.3. Database Search and Analysis

Spectronaut 13 (Biognosys AG) was used on all raw DDA data files to produce an initial list of protein targets filtered to a 1% protein-level false discovery rate (FDR). The files were searched against a database of Bos Taurus with 23,858 entries. DIA data were analyzed by the BGS Factory Setting (default) of Spectronaut 13, which uses the iRT peptides for retention time calibration. The decoy generation was set to “mutated”, and at the precursor and protein levels, 1% FDR was used. In short, all selected precursors that passed the filters were quantified. Except for the 3 least interfering ions, MS1 will remove all interfering fragment ions. The major group quantities were computed using the average of the top three filtered peptides that passed the 1% Q value cutoff. Following the Welch’s ANOVA Test, differentially expressed proteins were filtered if their *p*-value < 0.05 and fold change > 1.3.

### 2.5. Parallel Reaction Monitoring (PRM)

The protein expression levels measured by the DIA technology were validated using the PRM approach. SpectroDive 9.10 was used to analyze the raw data. The Q-value cutoff on the precursor was set at 1%. Protein amounts were calculated by taking the average of the filtered peptides.

### 2.6. Bioinformatics and Statistical Analysis

Gene Ontology (GO) annotations were performed using the Blast2 GO version 5 software to obtain a functional classification of the differentially abundant proteins. The function classification of EuKaryotic Orthologous Groups (KOGs) was accomplished through the phylogenetic classification of proteins encoded in complete genomes (NCBI, http://www.ncbi.nlm.nih.gov/, accessed on 7 September 2021). The pathways annotation of proteins was elucidated based on the Kyoto Encyclopedia of Genes and Genomes (KEGG) using the KOBAS (http://kobas.cbi.pku.edu.cn/, accessed on 15 October 2021). STRING v 11.5 software (http://string-db.org/, accessed on 12 December 2021) was applied to process the protein–protein interaction network.

R software was used to perform principal component analysis (PCA), partial least squares discrimination analysis (PLS-DA), hierarchical cluster analysis (HCA). Furthermore, Pearson correlation coefficient analysis between the differentially abundant proteins and meat quality was also completed using R software. The data of meat quality were analyzed by SPSS.20.0 software. PRM data was analyzed and graphed with the GraphPad Prism 9 software. Statistical analysis was made with the one-way analysis of variance (ANOVA) and Tukey’s multiple comparisons test at 5% of significance. All results were expressed as means ± standard deviations (SD) and *p* < 0.05 was considered significant.

## 3. Results and Discussion

### 3.1. Meat Quality of the Beef Samples

There was marked variation in meat quality from different frozen temperatures, and the main factors, including color, thawing loss, cooking loss, and shear force, are shown in Table 1. The results indicated that freezing significantly impacted the meat color, which corroborates the previous research findings [6]. Frozen/thawed samples of −12 °C group were shown to be lighter (higher L*, *p* < 0.05) than the fresh samples, which could be owing to myofibril contraction resulting in increased water flow onto the muscle surface and greater light reflection [10]. Additionally, with the decrease in the freezing temperature, L* values decreased gradually, with no significant difference among the frozen groups (*p* > 0.05). The a* values declined significantly in the frozen group muscles compared with the CON (*p* < 0.05). The oxidation of myoglobin to MetMb, which was accelerated by the high freezing temperatures, was linked to lowering a* levels [9]. This was consistent with the findings of Qian et al. [26], who confirmed the a* values deteriorated less under ultra-fast freezing conditions. Regarding the b* values, there was an increase in the frozen group muscle compared with the CON. This could be related to the degree of lipid oxidation, the denaturation of myoglobin, and metmyoglobin formation during the freezing process [27]. Freezing affected the degree of protein and lipid oxidation as well as the redox ability of enzymes [6]; low freezing temperatures could also limit color deterioration.

The water-holding capacity (WHC) was characterized as thawing loss and cooking loss in this study. As expected, a decreasing trend in thawing loss and cooking loss can be observed with the decrease in the freezing temperatures. The thawing loss of the −12 °C sample was significantly higher (*p* < 0.05) than that of the −18 °C, −38 °C, and −80 °C samples (Table 1), most likely due to the significant breakdown of the muscle fiber architecture and protein denaturation that occurred at −12 °C freezing temperatures. Therefore, meat with low cooking loss may have a better eating quality. The influence of different freezing temperatures on the cooking loss of the samples is presented in Table 1. As shown, the cooking loss was significantly lower in the CON group than in the frozen groups (*p* < 0.05). Moreover, the higher freezing temperatures (−12 °C and −18 °C) were associated with higher cooking losses. The shear force significantly decreased (*p* < 0.05) after freezing and it also exhibited similar trends to the WHC among the −12 °C, −18 °C, −38 °C, and −80 °C groups. This is also in good agreement with the results of Lagerstedt et al. [7]. They hypothesized that ice crystal formation and growth during freezing stresses muscle fibers, causing severe fiber damage, affecting the shear force and the WHC.

### 3.2. Protein Profiling in Fresh and Frozen Samples

**Figure 2:**
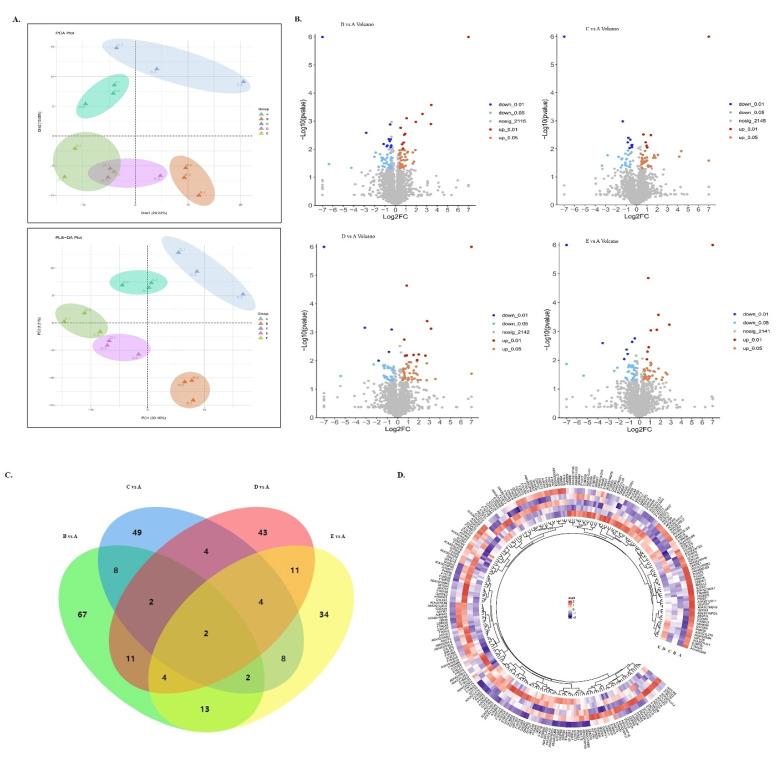
(**A**) PCA score plots and PLS-DA score plots between frozen treatments and CON. (**B**) Volcano plots showing differential abundance proteins (DAPs) in the beef muscle of −12 °C/CON, −18 °C/CON, −38 °C/CON, and −80 °C/CON comparisons. (**C**) Venn diagrams representing the overlap of DAPs from the −12 °C/CON, −18 °C/CON, −38 °C/CON, and −80 °C/CON comparisons. (**D**) Hierarchical cluster analysis of differential abundance proteins (DAPs) in the beef muscle of different treatments compared with CON. (In PCA score plots, PLS-DA score plots, and Venn diagrams, A represents CON; B represents −12 °C; C represents −18 °C; D represents −38 °C; and E represents −80 °C.)

DIA technology was used to investigate the protein profile changes of beef muscle in the fresh (CON) and frozen groups (−12 °C, −18 °C, −38 °C, and −80 °C). In general, a total of 32,416 precursors were obtained, which were matched to 32,005 unique peptides and 2287 protein groups with 1.0% FDR (Supplementary Tables S1 and S2). A total of 2610 proteins were used for quantitative analysis. To better visualize the aggregation and dispersion of the proteins among the four groups, PCA and PLS-DA were used in this study. Based on the first two principal components, there was a clear separation between both the CON and each treatment group (Figure 2A), which indicated that freezing could remarkably alter the protein expression level of the beef samples. Among all the identified proteins, 262 displayed significant differences in expression levels when filtered with a fold change > 1.3 and a *p*-value < 0.05 in −12 °C/CON, −18 °C/CON, −38 °C/CON, and −80 °C/CON, as shown in Supplementary Table S3. Following statistical analysis, volcano plots displayed the differentially abundant proteins (DAPs) between each frozen group and CON (Figure 2B). DAPs were found to be 109 between −12 °C and CON (59 downregulated and 50 upregulated), 79 between −18 °C and CON (43 downregulated and 36 upregulated), 81 between −38 °C and CON (37 downregulated and 44 upregulated), and 78 between −80 °C and CON (39 downregulated and 39 upregulated) (Figure 2B,C). More significant amounts of DAPs, in the comparison between the −12 °C/CON and the other three temperatures, indicated many more variations in the protein profile of the beef stored at −12 °C. These results were consistent with the quality changes.

The top ten most noticeably up- or downregulated proteins were CTTN, STAT3, EIF3G, THYN1, PDXP, SARS2, TBCC, and GAA. As shown in Supplementary Table S3, the most significantly downregulated protein was TBCC, which was found in the −80 °C/CON group, while SARS2 was the most significantly upregulated protein, which occurred at the −38 °C/CON condition. Moreover, CTTN (cortactin), a member of the actin-binding protein family that regulates the structure of the actin cytoskeleton, was the protein shared by the −12 °C, −38 °C, and −80 °C treatments. The changes in cortactin might affect the contraction of myofibrillar protein and further lead to quality deterioration [17]. Hierarchical cluster analysis (HCA) was performed to visually display the expression of the DAPs in each treatment. The dynamic changes of the DAPs were presented in Figure 2D. The change in the content of the differentially expressed proteins from high to low was represented by the color scale from red to purple. Colors in different freezing temperature groups were significantly different, confirming that the freezing temperature had the most significant influence on the DAP profile in the beef samples.

The top 10 highest abundance proteins among the fresh and frozen treatments included MYH1, TNNT3, MYH13, ENO3, TPM1, ACTN3, ATP2A1, PSMD7, ATP5F1B, and PGM1. As can be seen in Figure 2D, all the proteins occurred at −12 °C except for PSMD7, which changed in the −80 °C group. Myosin is an actin-based motor protein that uses ATP hydrolysis energy to move actin filaments and generate force [28]. It is essential for muscle organization, structure, and function. The structural integrity of muscle fibers may be damaged if a heavy chain of myosin is oxidized [29]. Myosin-1 (MYH1), a myosin family member that can connect to actin filaments and cellular membranes at the same time and is involved in muscle contraction [12], was found to be the most abundant protein in this study. Gagaoua et al. [30] also reported MYH1 in their review of beef color biomarkers. Myosin heavy chain (MYH13), a TRAFAC class myosin–kinesin ATPase superfamily member, plays a critical role in muscle structure and calcium ion binding [31]. Troponin T3 (TNNT3) was also reported as a structural protein and significantly associated with meat tenderness [32]. In addition, tropomyosin alpha-1 chain (TPM1) and alpha-actinin-3 (ACTN3), known as the binding protein, played a central role in muscle contraction and calcium ion binding [14]. According to the previous studies, a few remaining proteins were identified as metabolic enzymes and participated in the glycolysis (ENO3, PGM1), energy metabolism (ATP5F1B), the regulation of calcium ion (ATP2A1), and cellular processes such as cycle progression and apoptosis [20].

### 3.3. GO, KOG, and KEGG Pathway Analysis of DAPs

**Figure 3:**
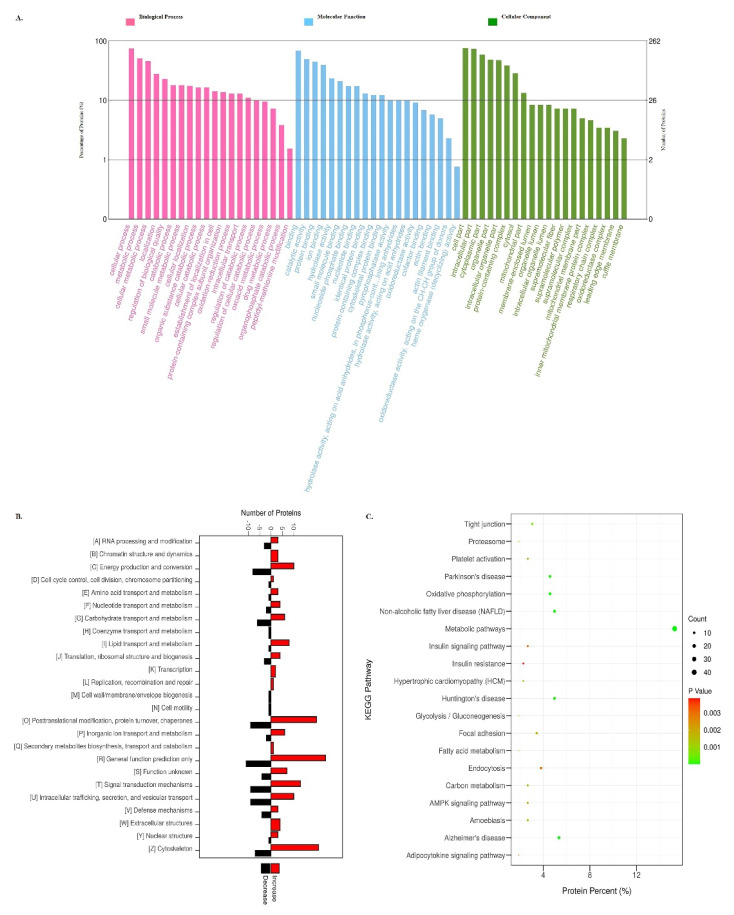
(**A**) Gene ontology (GO) classification of differentially expressed proteins in frozen beef vs. CON group. (**B**) KOG analysis of DAPs (x-axis displays DAPs count, y-axis displays KOG terms) in frozen beef vs. CON group. (**C**) KEGG pathway of DAPs in frozen beef vs. CON group.

GO analysis was applied to investigate the potential functions of 262 DAPs under various freezing temperature conditions. Based on the analysis, there were 911 items of biological process, 242 items of molecular function, and 150 items of cellular component between the frozen group samples and the CON comparison (Supplementary Table S4). Figure 3A presents the top 20 enriched GO terms for these DAPs. In the biological process, the DAPs were mainly distributed in the cellular (74.81%), metabolic process (50.38%), cellular metabolic (45.80%), localization (27.86%), and regulation of biological quality (22.90%). Proteins with the cellular component were mainly located in the cell part (75.95%), intracellular (73.28%), cytoplasmic (58.40%), organelle (48.47%), and intracellular organelle part (47.71%). Regarding the molecular function, most DAPs were involved in the binding and activity process, mainly including binding (68.70%), protein binding (44.27%), cytoskeletal protein binding (12.21%), catalytic activity (49.24%), hydrolase activity (23.28%), and so on. These results were comparable to those obtained by Jia et al. for Hengshan goat during freezing storage [20]. This suggests that freezing significantly affects protein function. In fact, there were fewer GO change terms in the −18 °C/CON, −38 °C/CON, and −80 °C/CON groups (649, 778, and 697 categories, respectively) than in the −12 °C/CON (888 categories) (Supplementary Table S5), implying that lower freezing temperatures had less of an impact on the protein functions.

As shown in Figure 3B, the KOG database was used to annotate and predict the functional classification of differential proteins based on sequence similarity. Notably, a total of 25 categories were identified based on the statistical analysis. The number of upregulated proteins in energy production and conversion, amino acid and lipid transport metabolism, protein turnover, chaperones, general function prediction only, and cytoskeleton were higher than the downregulated proteins. Additionally, the number of proteins involved in the chromatin structure and dynamics, transcription, recombination and repair, secondary metabolites biosynthesis, and extracellular exhibited increasing trends, whereas the coenzyme transport and metabolism, membrane biogenesis, and cell motility showed decreasing trends. The top three function classes were the general function prediction only, cytoskeleton, and post-translational modification, protein turnover, and chaperones. This was consistent with the findings of Song Yu et al. [16], indicating that, regardless of whether meat is processed at a low or high temperature, the protein function will be affected, particularly its structural function.

KEGG pathway enrichment was performed in this study to further analyze the biological processes and functions of the DAPs in response to the frozen treatment. Among the 262 DAPs, a total of 210 KEGG pathways were matched, and those with *p* < 0.05 were regarded as significantly enriched pathways (Supplementary Table S6). Compared with the CON, the 20 pathways with the highest *p*-values are presented in Figure 3C. DAPs were shown to be enriched in the metabolism (metabolic, oxidative phosphorylation, fatty acid metabolism, carbon metabolism, and glycolysis/gluconeogenesis), genetic information processing (proteasome), environmental information processing (AMPK signaling pathway), and cellular processes (tight junction and focal adhesion pathway). In-depth analysis indicated that the pathways with the highest *p*-values in the −12 °C/CON comparison were also mainly metabolic pathways, followed by oxidative phosphorylation, tight junction, and glycolysis/gluconeogenesis. As shown in Supplementary Table S6, seven proteins (SDHA, NDUFA13, NDUFA6, NDUFB9, NDUFS7, ATP5F1B, and NDUFA11) participated in the oxidative phosphorylation under the −12 °C/CON group. Previous studies confirmed that oxidative phosphorylation is the main pathway that produces ATP in mitochondria and is related to meat quality [33]. The downregulation of these proteins shown in the −12°C group might be the main reason for their significantly lower quality compared to −18 °C, −38 °C, and −80 °C. In addition, TECR, ACADVL, FASN, and HADH were identified in the −12 °C group, and these four proteins participated in fatty acid metabolism, fatty acid elongation, degradation, and biosynthesis pathways. The metabolic pathway at −18 °C was essentially the same as −12 °C, focusing on the structure and energy metabolism pathways, such as endocytosis, the regulation of actin cytoskeleton, focal adhesion, and glycolysis/gluconeogenesis (Table S6). Jia et al. [20] and Hou et al. [15] reported that adhesion maintained the integrity of muscle cells and influenced the formation of drip channels, which could affect the quality of the meat. Redox stability myoglobin is significantly affected by glycolysis [11]. This may account for the significant difference in the a* value between the −12 °C group and the other groups. The tight junction and the AMPK signaling pathways were the primary pathways at −38 °C, but the AMPK and FoxO signaling pathways dominated at −80 °C. PRKAG1, FASN, PRKAB1, and MAPK9 were upregulated in the −80 °C group and participated in these two signaling pathways, which inhibited the energy-consuming biosynthetic pathways while activating the ATP-producing catabolic pathways, regulating oxidative phosphorylation, glucose metabolism, apoptosis, and oxidative stress [34].

### 3.4. Protein–Protein Interaction Analysis

**Figure 4:**
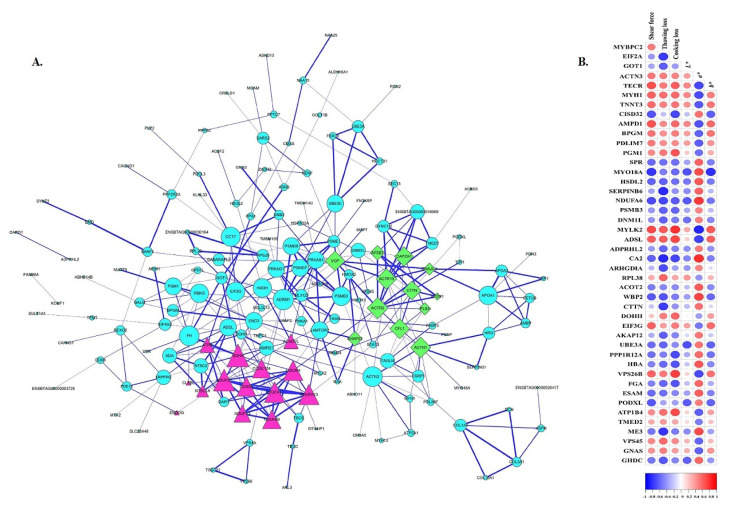
(**A**) Protein–protein interaction (PPI) network of the total DAPs. (**B**) A diagram of proteins correlated with at least one quality indicator (r > 0.60; *p* < 0.05).

String 11.5 was used for the protein–protein interaction (PPI) analysis to comprehensively understand the functions and relationships of these differentially expressed proteins in four comparable groups. As seen in Figure 4A, the PPI enrichment *p*-value < 1.0 × 10^16^ and the local clustering coefficient was 0.397. Most of these proteins attended the metabolic pathways in our study. Meanwhile, the close connection of NDUFA11 with COX5A, NDUFA13, NDUFV3, NDUFB9, NDUFS7, UQCRH, SDHA, and ATP6 indicated that these proteins regulated the oxidative phosphorylation of thawed muscle together, whereas proteins (CTTN, ACTN1, and ACTR2) involved in the tight junction pathway interacted. Yu et al. [12] reported that the protein subunits of complex I, complex II, and complex IV of the mitochondrial electron transfer chain might well be linked to beef discoloration. Proteins (ADRM1, PSME1, PSMB3, PSMD8, and PSMD7) participated in the proteasome pathway. In addition, proteins (ACADL, FASN, HADH, ACADVL, and TECR) that were implicated in fatty acid metabolism interacted strongly with each other. However, it can be noticed that the changes of the main functions and metabolic pathways (such as oxidoreductase activity, cytoskeletal protein binding in the MF, and the oxidative phosphorylation pathway) of all the DAPs at −12 °C and −18 °C were greater than that at −38 °C and −80 °C. This result was consistent with the GO, KOG, and KEGG pathway analysis results.

### 3.5. Correlations between DAPs and Meat Quality 

Pearson correlation analysis was used to examine the possible connection between 262 DAPs and meat quality attributes (thawing loss, cooking loss, shear force, and color) under various conditions (CON, −12 °C, −18 °C, −38 °C and −80 °C). Among them, 90 overlapping DAPs showed a significant (*p* < 0.05) correlation with at least one quality indicator (Supplementary Table S7). The proteins with r > 0.60 and *p* < 0.05 were shown in Figure 4B.

#### 3.5.1. Structure Proteins

Structure proteins (MYH, TNNT, ACTN3, and MYBP) were found to be the most abundant proteins in beef that had a positive or negative correlation with the meat quality [12,14,35,36]. Similar results were obtained in our work. There were nine-, seven-, three-, and three-structure proteins associated with the color, shear force, thawing loss, and cooking loss, respectively (Figure 4B). Most of these proteins were involved in the tight junction, actin cytoskeleton regulation, and focal adhesion pathways. This is consistent with previous research [4], which suggested that protein contractile properties may influence the correlation between protein and quality traits. In contrast to previous studies that found structural proteins more related to tenderness, they were primarily associated with color in this study. Holman et al. [10] also confirmed that muscle fiber shrinkage impacts the achromatic light scattering properties. As shown in Supplementary Table S7, myosin-1(MYH1) presented a negative correlation with the a* values (r = −0.708; *p* = 0.003) and was positively related to the shear force (r = 0.614; *p* = 0.015). This result was in line with the research of Yu et al. [12], who reported that MYH1 was negatively correlated with the a* value of Holstein beef muscle during postmortem storage. According to Shi et al. [18], myosin is important in muscle structure and function, as well as muscle color at low storage temperatures. MYBPC2, a myosin-binding protein involved in the regulation of muscle contraction, had a negative relationship with the a* values (r = −0.603; *p* = 0.017). It was reported that MYBPH is also a myosin-binding protein which had been linked to meat color in frozen Japanese pufferfish [37]. Actinin alpha 3 (ACTN3) and Troponin T3 (TNNT3) were also observed to have a significant negative correlation with the a* values (r = −0.626; *p* = 0.013). It was similar to the result of Gagaoua et al. [36]. However, myosin XVIIIA (MYO18A) was identified as a new quality-related protein in the current study. There was a high negative correlation with the b* values (r = −0.806; *p* = 2.874 × 10^−4^) and L* values (r = −0.673; *p* = 0.006), and a positive relationship with the a* values (r = 0.693; *p* = 0.004) in −12 °C group, with a downregulation trend. Simultaneously, MYO18A was found to be significantly negatively related to the shear force (r = −0.788; *p* = 4.892 × 10^−4^) and cooking loss (r = −0.581; *p* = 0.023). MYO18A is an unconventional myosin that has been linked to a number of cellular processes, including retrograde actin treadmilling and focal adhesion function. A previous study shed light on MYO18A’s abundant phosphorylation and other post-translational modifications [28]. This could be the primary reason why MYO18A has such a strong influence on meat quality. Cortactin (CTTN) is a cytoskeletal actin-binding regulatory protein that regulates the actin cytoskeleton structure. It had a noteworthy negative relationship with thawing loss (r = −0.756; *p* = 0.001). CTTN expression levels in this study were twice as high at −38 °C and −80 °C as they were at −12 °C, indicating that the freezing conditions affect the cytoskeleton, with −12 °C being especially harsh. As a binding protein, CDGSH iron–sulfur domain-containing protein 2 (CISD2) was proven to have a strong correlation with cooking loss (r = −0.712; *p* = 0.003) and shear force (r = −0.668; *p* = 0.006), respectively. The majority of these proteins are found at −12 °C, indicating that −12 °C has a greater impact on the degradation and aggregation of structural proteins, which destroys the cytoskeletal integrity, thus affecting quality.

#### 3.5.2. Enzymes

Variations of enzymes are tightly associated with glycolysis/gluconeogenesis, oxidative phosphorylation, and the metabolic pathway of frozen samples under different temperatures. To some extent, the glycolytic content and rate of glycogenolysis affect the meat quality [15]. Phosphoglycerate mutase (BPGM) and phosphoglucomutase-1 (PGM1) are enzymes involved in glycolysis/gluconeogenesis metabolism. These two proteins were found to be differentially expressed between −12 °C and the CON. BPGM was discovered to be upregulated and showed a significant negative relationship with the a* values (r = −0.613; *p* =0.015). This result was consistent with a previous study in which Faustman et al. [38] discovered that an increase in glycolytic enzymes harmed myoglobin’s redox stability. Contrary to expectations, PGM1 was not only associated with color but also had a positive correlation with cooking loss (r = 0.665; *p* = 0.007) in this investigation. Glycogen is gradually converted into lactic acid during glycolysis, resulting in a drop in pH, which affects protein stability and muscle water retention [12].

NADH dehydrogenase is an enzyme that transfers electrons from NADH to the respiratory chain in the mitochondrial membrane [20]. According to the findings of this study, NDUFA6 was an accessory subunit of NADH dehydrogenase (complex I) that participated in the oxidative phosphorylation pathway and exhibited a correlation with meat quality. NDUFA6 showed a strong positive relationship with the a* values (r = 0.773; *p* = 7.335 × 10^−4^) and a negative association with the L* values (r = −0.644; *p* = 0.009), cooking loss (r = −0.646; *p* = 0.009), thawing loss (r = −0.717; *p* = 0.003), and shear force (r = −0.7; *p* = 0.004). This was in accordance with the report by Yu et al. [12]. NADH is primarily concentrated in downregulated protein and promotes the reduction of metmyoglobin, which improves the stability of meat color in muscle. Meat in the CON group was redder than in the other groups, probably due to the interaction of phosphorylated mitochondrial proteins and glycolytic enzymes in the energy metabolism [39]. On the other hand, a higher abundance in the −12 °C group indicated a lower tenderness, which is consistent with the shear force result. Myosin light chain kinase 2 (MYLK2) as a new biomarker, there was a negative relationship with the a* values (r = −0.845; *p* = 7.381 × 10^−5^), and a positive correlation with the b* values (r = 0.644; *p* = 0.009), thawing loss (r = 0.648; *p* = 0.009), cooking loss (r = 0.791; *p* =4.408 × 10^−4^), and shear force (r = 0.709; *p* = 0.003). This could be related to the phosphorylation of a specific serine in a myosin light chain’s N-terminus. The upregulated phosphorylated proteins may have slowed the degradation of the protein structure and thus contributed to the WHC [39].

The mitochondrial NADP (+)-dependent malic enzyme 3 (ME3) is a key malate dehydrogenase that is primarily responsible for catalyzing the reversible oxidative decarboxylation of malate to form pyruvate. Most of the previous research has focused on the relationship between MDH or LDH and muscle quality. However, this study revealed a positive correlation with the a* values (r = 0.704; *p* = 0.003), as well as a negative relationship with the thawing loss (r = −0.783; *p* =5.509 × 10^−4^). Gill et al. [40] hypothesized that the change in the quality was caused by ME-catalyzed pyruvate conversion. SERPINB6 is a downregulated shared protein found in four frozen groups that are involved in the regulation of serpin peptidase. It displayed a close relationship with the thawing loss (r = −0.825; *p* =1.514 × 10^−4^). Lopez-Pedrouso et al. [41] discovered SERPINB6 to be highly correlated with IMF, but little is known about its connection to meat quality. Carbonic anhydrase 2 was found to be downregulated in frozen groups at −12 °C and −18 °C. CA2 has long been known to promote the rapid conversion of glycolytic intermediates to oxaloacetate and citrate, thereby increasing ATP synthesis [42]. It had a high positive association with the a* values (r = 0.811; *p* = 2.4 × 10^−4^), and a negative relationship with the thawing loss (r = −0.832; *p* = 1.181 × 10^−4^) and cooking loss (r = −0.723; *p* = 0.02) in our study. Proteasome subunit beta type-3 (PSMB3) was discovered in the −12 °C group. It is a component of the 20S proteasome that is involved in the ATP-dependent degradation of ubiquitinated proteins while closely binding to ACTN3. It had a negative correlation with thawing (r = −0.616; *p* = 0.015). Aspartate aminotransferase (GOT1), adenylosuccinate lyase (ADSL), and very-long-chain enoyl-CoA reductase (TECR) were mainly involved in the metabolism of amino acids and fatty acids. GOT1 showed a negative relation to the thawing loss (r = −0.632; *p* = 0.011). TECR and ADSL were positively correlated with the shear force (r = 0.689; *p* = 0.005), cooking loss (r = 0.631; *p* = 0.012) and was negatively related to the a* value (r = −0.719; *p* = 0.003). The changes in the protein expression may be related to the oxidation reaction that occurs during the freezing process. Protein phosphatase 1 regulatory subunit (PPP1R12A) is concerned with skeletal integrity and the muscle contractile function. There was a negative relationship with the cooking loss (r = −0.701; *p* = 0.004). Xia et al. [43] reported that PPP1R12A was related to meat tenderness. The results of this study agree with it. Moreover, this protein was found to have a negative correlation with the shear force.

#### 3.5.3. Other Proteins

Eukaryotic initiation factor 2A (EIF2A) is a multimeric protein. According to the previous report, EIF2A is required for the maintenance of the rate-limiting step in mRNA translation and is in charge of binding GTP and transferring Met-tRNA to the 40S ribosomal subunit [44]. It was found to have a significant negative relationship with the thawing loss (r = −0.768; *p* = 8.336 × 10^−4^) and cooking loss (r = −0.682; *p* = 0.05), as well as a positive relation with the a* value (r = 0.629; *p* = 0.012) in this study. WW domain-binding protein 2 (WBP2) was identified to have a significant negative relationship with the shear force (r = −0.646; *p* = 0.009) and thawing loss (r = −0.664; *p* = 0.007), and a positive correlation with the a* value (r = 0.734; *p* = 0.002) in this study. WBP2 was a binding partner of the WW domain protein, interacting with several WW-domain-containing proteins, and regulating cytoskeletal activity; this might also explain its association with the shear force and thawing loss [45]. Protein1B4 (ATP1B4) was involved in muscle contraction and had a positive relationship to the cooking loss (r = 0.707; *p* = 0.003). Rho GDP-dissociation inhibitor-1(ARHGDIA) showed a negative relation to the thawing loss (r = −0.697; *p* = 0.004). It might be used as a new potential biomarker for the frozen bovine *Longissimus thoracic* muscle at −12 °C, −38 °C, and −80 °C. However, the relationship between these proteins and the meat quality is not well understood, and the molecular mechanism requires further investigation.

### 3.6. Validation of DAPs by PRM

**Figure 5:**
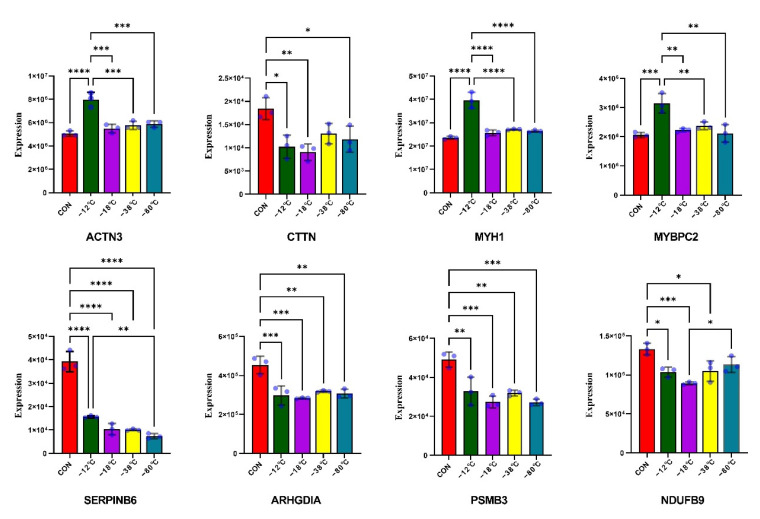
Expression profiles of the proteins in different freezing temperatures were validated by parallel reaction monitoring (PRM). The results were represented as mean ± SD from three experiments. Asterisks represent levels of significance (* *p* < 0.05; ** *p* < 0.01; *** *p* < 0.001; **** *p* < 0.0001).

MYH1, ACTN3, CTTN, MYBPC2, ARHGDIA, PSMB3, NDUFB9, and SERPINB6 were selected for RPM analyses to validate the DIA proteomics results. As shown in Figure 5A, the expression levels of SERPINB6 (serpin B6) and ACTN3 (alpha-actinin-3) increased in the four frozen groups compared to the CON, whereas the other six proteins presented decreasing expression levels. Thus, the PRM results for these proteins were consistent with those obtained from the DIA analysis, indicating that the DIA proteomic analysis approach is trustworthy.

## 4. Conclusions

In summary, the changes in the muscle proteomics profiles and biological information at different temperatures were understood in this study. Compared with the CON group, a total of 262 proteins were identified as DAPs, of which 109, 79, 81, and 78 proteins were in overabundance in −12 °C/CON, −18 °C/CON, −38 °C/CON, and −80 °C/CON, respectively. The bioinformatics analysis revealed that most of the DAPs were involved in the protein binding and catalytic activity of GO items, and participated in the oxidative phosphorylation, glycolysis/gluconeogenesis, tight junction, and focal adhesion pathways. Furthermore, the changes in the meat quality were more severe in the −12 °C condition than in the −18 °C, −38 °C, and −80 °C. As expected, −80 °C had better freezing quality traits. Correlation analysis between the DAPs and the quality traits showed that 90 proteins were significantly correlated with the color (L*, a*, and b*), thawing loss, cooking loss, and shear fore of the frozen beef. Among them, 17 closely related proteins could be potential biomarkers for frozen beef quality. The putative biomarkers identified in this study should be evaluated and validated in a larger group of animals to predict the frozen beef quality.

## Figures and Tables

**Table 1:** Quality characteristics of beef in fresh control (CON) and different frozen treatments (−12 °C, −18 °C, −38 °C, −80 °C).

Attribute	CON	−12 °C	−18 °C	−38 °C	−80 °C	*p*-Value
L*	38.05 ± 0.51 b	40.06 ± 0.70 a	39.59 ± 1.81 ab	39.17 ± 0.90 ab	39.04 ± 0.79 ab	0.034
a*	15.38 ± 0.71 a	12.94 ± 0.34 c	13.40 ± 0.18 c	14.42 ± 0.28 b	14.47 ± 0.24 b	<0.001
b*	11.66 ± 1.38 c	13.19 ± 0.23 a	12.78 ± 0.39 ab	12.03 ± 0.12 bc	11.75 ± 0.18 bc	0.001
Thawing Loss/%	–	10.50 ± 0.84 a	8.83 ± 0.75 b	7.67 ± 0.52 bc	7.17 ± 0.7 c	<0.001
Cooking Loss/%	28.28 ± 2.12 c	34.0 ± 26 a	32.33 ± 2.34 ab	30.33 ± 3.14 abc	29.83 ± 172 bc	0.001
Shear Force/kg	7.81 ± 0.69 b	10.72 ± 0.52 a	9.94 ± 0.64 a	8.36 ± 0.57 b	8.22 ± 0.58 b	<0.001

All the values are presented as means ± SD, *n* = 6. Different letters, a–c, represent the significance between the different treatments. *p* < 0.05 was considered significant.

## Data Availability

The data presented in this study are available upon request from the corresponding author.

## Supplementary Materials

The following supporting information can be downloaded at: https://www.mdpi.com/article/10.3390/foods11121791/s1. Supporting Table S1: A list of unique peptides identified in the current study using DIA technology.; Supporting Table S2: A list of protein groups identified in the current study using DIA technology; Supporting Table S3: A list of differentially abundant proteins (DAPs) in the current study using DIA technology; Supporting Table S4: Detailed explanations for the GO enrichment of differentially abundant proteins (DAPs) between frozen groups and CON; Supporting Table S5: Detailed explanations for the GO enrichment of differentially abundant proteins (DAPs) of each comparison group (−12 °C/CON, −18 °C/CON, −38 °C/CON, and −80 °C/CON); Supporting Table S6: Detailed explanations for the KEGG pathway enrichment of differentially abundant proteins (DAPs) between frozen groups and CON; Supporting Table S7: Detailed explanations for the correlation analysis results between DAPs and meat quality.
